# Impact of Continuous Kidney Replacement Therapy and Hemoadsorption with CytoSorb on Antimicrobial Drug Removal in Critically Ill Children with Septic Shock: A Single-Center Prospective Study on a Pediatric Cohort

**DOI:** 10.3390/antibiotics12091395

**Published:** 2023-08-31

**Authors:** Gabriella Bottari, Bianca Maria Goffredo, Marco Marano, Cristina Maccarrone, Raffaele Simeoli, Giuseppe Bianco, Leonardo Vallesi, Joseph Charles Charlie Beetham, Anna Teresa Mazzeo, Andrea Cappoli, Sara Cairoli, Raffaella Labbadia, Corrado Cecchetti, Paola Bernaschi, Tiziana Corsetti, Santo Morabito, Fabio Silvio Taccone, Isabella Guzzo

**Affiliations:** 1 Pediatric Intensive Care Unit, Bambino Gesù Children’s Hospital, IRCCS, 00165 Rome, Italy; marco.marano@opbg.net (M.M.); charlie.beetham@opbg.net (J.C.C.B.); corrado.cecchetti@opbg.net (C.C.); 2 Division of Metabolic Diseases and Drug Biology, Bambino Gesù Children’s Hospital, IRCSS, 00165 Rome, Italy; bianca.goffredo@opbg.net (B.M.G.); raffaele.simeoli@opbg.net (R.S.); sara.cairoli@opbg.net (S.C.); 3 Anesthesia and Intensive Care Department of Human Pathology, University of Messina, 98158 Messina, Italy; cristina.maccarrone@asp.messina.it (C.M.); mazzeo.anna@unime.it (A.T.M.); 4 Hospital Pharmacy Unit, Bambino Gesù Children’s Hospital, IRCCS, 00165 Rome, Italy; giuseppe2.bianco@opbg.net (G.B.); leonardo.vallesi@opbg.net (L.V.); tiziana.corsetti@opbg.net (T.C.); 5 Division of Nephrology and Dialysis, Bambino Gesù Children’s Hospital, IRCCS, 00165 Rome, Italy; andrea.cappoli@opbg.net (A.C.); raffaella.labbadia@opbg.net (R.L.); isabella.guzzo@opbg.net (I.G.); 6 Microbiology and Diagnostic Immunology Unit, Department of Diagnostic and Laboratory Medicine, Bambino Gesù Children’s Hospital, IRCCS, 00165 Rome, Italy; paola.bernaschi@opbg.net; 7 Hemodialysis Unit, Department of Internal Medicine and Medical Specialties, Policlinico Umberto I, 00161 Rome, Italy; santo.morabito@uniroma1.it; 8 Department of Intensive Care, Hopital Universitaire de Bruxelles (HUB), Université Libre de Bruxelles (ULB), 1050 Brussels, Belgium; fabio.taccone@ulb.ac.be

**Keywords:** therapeutic drug monitoring (TDM), extracorporeal therapies, CKRT, hemoadsorption, CytoSorb, antimicrobials, clearance, mass removal

## Abstract

**Background:** Extracorporeal therapies (ET) are increasingly used in pediatric settings as adjuvant therapeutic strategies for overwhelming inflammatory conditions. Although these treatments seem to be effective for removing inflammatory mediators, their influence on antimicrobials pharmacokinetic should not be neglected. **Methods:** A prospective observational study of children admitted to the pediatric intensive care unit (PICU) with a diagnosis of sepsis/septic shock. All critically ill children received hemoadsorption treatment with CytoSorb (CS) in combination with CKRT. Therapeutic drug monitoring has been performed on 10 critically ill children, testing four antimicrobial molecules: meropenem, ceftazidime, amikacin and levofloxacin. In order to evaluate the total and isolated CKRT and CS contributions to antibiotic removal, blood samples at each circuit point (post-hemofilter, post-CS and in the effluent line) were performed. Therefore, the clearance and mass Removal (MR) of the hemofilter and CS were calculated. **Results:** Our preliminary report describes a different impact of CS on these target drugs removal: CS clearance was low for amikacine (6–12%), moderate for ceftazidime (43%) and moderate to high for levofloxacine (52–72%). Higher MR and clearance were observed with CKRT compared to CS. To the best of our knowledge, this is the first report regarding pharmacokinetic dynamics in critically ill children treated with CKRT and CS for septic shock.

## 1. Background

Extracorporeal blood purification techniques (ETs) such as hemodialysis and hemofiltration have been used successfully for several decades with the aim of replacing renal function in critically ill patients with kidney failure. In recent years, the concept of extracorporeal organ support (ECOS) has emerged to describe all forms of therapies where blood is extracted from the body and processed in different circuits with specific devices and techniques [1].

Critical status, sepsis and ET are all factors that may influence the ideal pharmacokinetic/pharmacodynamic (PK/PD) targets of many drugs, including antimicrobial agents [2,3,4,5,6]. The potential impact of ET on concomitantly administered drugs is not fully understood, and the lack of data limits the availability of evidence-based recommendations for antibiotic (AB) dosing. Moreover, there are several age-related factors that influence the PK properties of drugs administered to pediatric patients.

Therefore, the “one dose fits all” approach is clearly unfeasible, and the implementation of a tailored approach in critical pediatric patients, especially if using ETs, may play a key role. To achieve this aim, therapeutic drug monitoring (TDM) represents a useful tool that could help intensive care physicians to adjust drug dosing in order to reach specific PK/PD targets [7]. To date, TDM seems to be the only safe and effective way to optimize antimicrobials treatments in critically ill patients. In fact, this approach could reduce the development of microbial resistance avoiding suboptimal antibiotics’ plasma concentration [8].

CytoSorb^®^ (CS) (CytoSorbents Corporation, Princeton, NJ, USA) is a novel synthetic hemoadsorption device that was approved by the European Union (conformité européenne—CE) for extracorporeal cytokine removal in 2011 [9]. CS cartridges can be easily integrated in extra-corporeal circulation circuits (Continuous Kidney Replacement Therapy—CKRT, Extracorporeal Membrane Oxygenation—ECMO, Cardio-Pulmonary Bypass—CPB), and can be used in a stand-alone modality (hemoadsorption). The cartridge contains biocompatible polystyrene divinylbenzene copolymer beads capable of removing molecules of medium molecular weight (according to their size exclusion mechanism) by using a combination of hydrophobic or ionic interactions as well as hydrogen bonding [9,10]. In pediatric settings, the device has shown an excellent safety profile and potential beneficial clinical effects as adjuvant therapy, helping to control overwhelming inflammatory conditions [11,12]. However, one critical issue that should be taken into account when administering anti-microbial therapies alongside to CS cartridge utilization is the augmented drug clearance, which exposes patients to the risk of poor clinical outcomes and the development of AB resistance as a result of AB sub-therapeutic levels. Actually, no data are available on the effect of CS on AB dosage in the pediatric field [13,14]. The aim of this monocentric study was to evaluate the impact of CS on the plasma levels of different ABs commonly used in a pediatric intensive care unit (PICU).

## 2. Results

We enrolled 10 pediatric patients who received hemoadsorption with CS and CKRT between October 2021 and March 2023. In Table 1, the demographic and clinical characteristics on admission, including etiology and source of infections, are reported.

### 2.1. CytoSorb and CKRT Clearance and Mass Removal

In Table 2, the hemofilter and CS mean values ± standard deviation (SD) for clearance and mass removal are reported. The mean clearance and mass removal for the antimicrobials analyzed in this study positively indicated drug removal by both devices, except for meropenem. In fact, the CS clearance and mass removal mean values for meropenem were both negative (Table 2). The average clearance was higher than 2 L/h only for levofloxacin. The mean values of mass removal and clearance showed a lower impact of CS compared to just the hemofilter, while levofloxacin clearance and mass removal values were significantly affected by CS at both the Cmin and Cmax levels.

**Figure 1:**
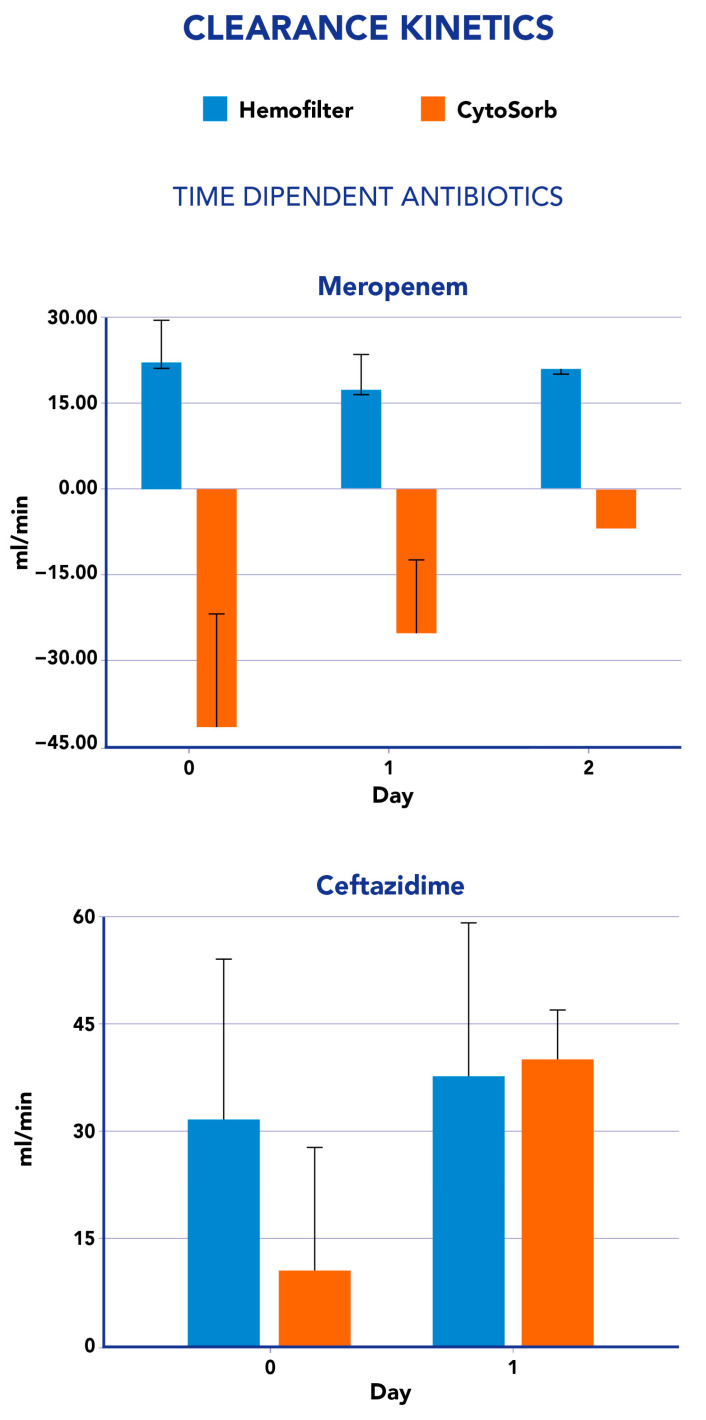
Clearance values for time-dependent antibiotics analyzed in this study (Meropenem and Ceftazidime). Blue bars refer to hemofilter clearance and orange bars to the CytoSorb clearance. *y*-axis: antibiotic clearance (mL/min); *x*-axis time points of the day antibiotic’s measurements.

**Figure 2:**
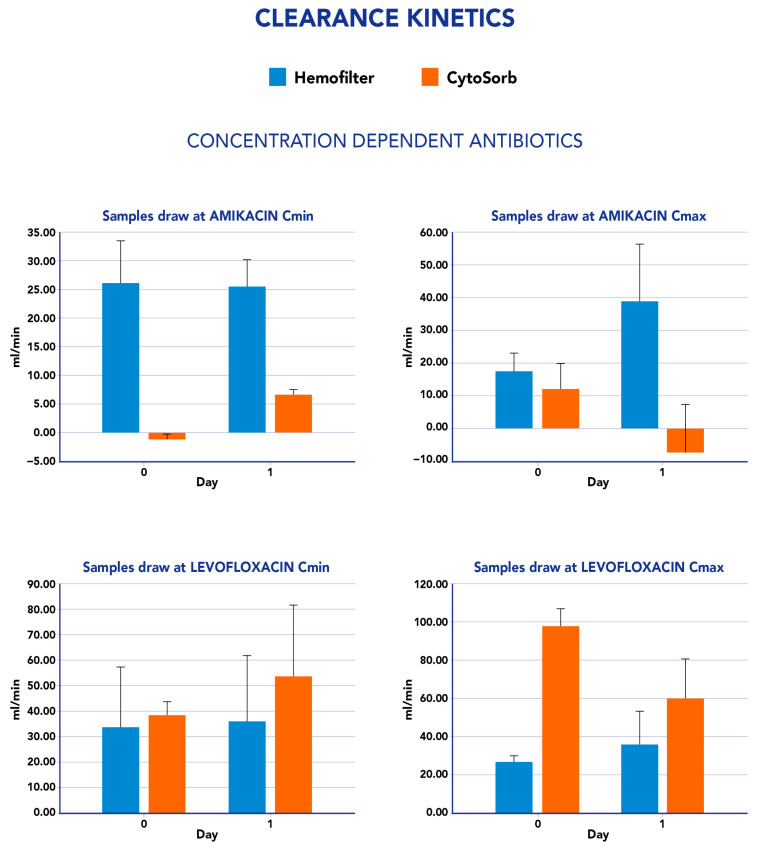
Clearance values for concentration-dependent antibiotics analyzed in this study (Amikacine and Levofloxacine). Blue bars refer to hemofilter clearance and orange bars to the CytoSorb clearance. *y*-axis: antibiotic clearance (mL/min); *x*-axis: time points of the day of antibiotic measurements.

The clearance values measured for both CS and CKRT were not constant during the study period. In fact, by analyzing the impact of CS on ET clearance, we observed an increase, in most cases, although meropenem constantly showed negative values (Figure 1). Similarly, CS-mediated clearance for ceftazidime showed an increase from the first day to the second day of the study period (Figure 1). For the concentration-dependent antibiotics amikacin and levofloxacin, we observed an increase in the clearance value at Cmin from the first to the second day, whereas a reduction was reported in the same period at Cmax measurements (Figure 2). We found statistically significant differences in clearance and mass removal between hemofilter and CS for meropenem on day 0—CL (*p* 0.009), MR (*p* 0.012), and on day 2—CL (*p* 0.021) and MR (*p* 0.026). For ceftazidime, we found a significant difference in MR (*p* 0.0495) on day 1. Regarding the concentration-dependent antibiotics, only amikacine CL at C*min* showed a significant difference between CKRT and CS (*p* 0.049). All the other evaluations at different time points for all antimicrobials tested resulted in no significant differences.

Supplementary Figure S1 reports the impact of the hemofilter on CL_ET_ for each antibiotic. CS was associated with an increase in the total clearance for all tested drugs except for meropenem (−57%). The impact was considered low for amikacine (6–12%), moderate for ceftazidime (43%) and moderate to high for levofloxacine (52–72%) [2].

### 2.2. Clinical Outcomes and AUC during ET Treatments

**Figure 3:**
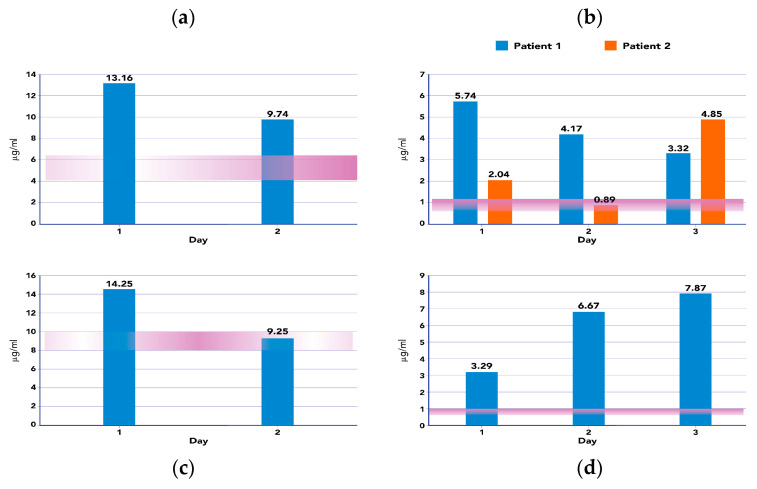
Graphical representation of the relationship between pharmacodynamic target attainment (PTA) (red bars) calculated on the basis of MIC and blood concentration (mcg/mL) of each antibiotic tested during the study (blue and orange bars). (**a**) Ceftazidime MIC for *Klebsiella pneumoniae*; (**b**) meropenem for Klebsiella pneumonia in two different patients (blue bars—patient 1 and orange bars—patient 2); (**c**) amikacin for *Klebsiella pneumonia*; (**d**) meropenem for *Streptococcal pneumonia*.

We observed a new onset of infections in 3 out of 10 patients by day 7 and in 1 out of 10 patients after 14 days. In our cohort, the median PICU length of stay was 11.5 days (8.75–20.25) and the PICU and 28-day mortality was 50%. A positive microbiological culture was observed in 5 out of 10 patients (including 4 patients with a positive hemoculture and 1 patient with a positive bronco-alveolar culture). As shown in Figure 3 the AB plasma levels achieved the pharmacodynamic target attainment (PTA) at all time points of the study period on the basis of the minimum inhibitory concentration (MIC) found. For patients with suspected septic shock and a negative culture, we evaluated the hypothetical PTA on the basis of the clinical scenario, antibiotic treatment and EUCAST breakpoint MIC values. As reported in Supplementary Table S1, antibiotic plasma levels were adequate enough to reach the PK/PD targets used in this study [15].

## 3. Discussion

In this study, we report for the first time an assessment of the PK/PD alteration due to CS and CKRT treatment in pediatric critically ill patients, considering the individual contributions from the different ETs used. In particular, we focused on four frequently used ABs with different PK/PD characteristics. Our data confirm the insignificant in vivo removal of meropenem by the CS cartridge, as previously reported [16]. Furthermore, we obtained a negative value of CS clearance for meropenem, suggesting an increase in AB levels at the CS outflow as a consequence of potential desorption. This mechanism has already been advocated by Schneider et al., particularly for beta-lactams [17]. The variation over the time of the desorption found in our study could be related to the nonlinear kinetics of the cartridge’s saturation during the 24 h of hemoadsorption therapy and could be explained by a saturation of the drug binding site on the absorber surface in the first 12 h and, after an adsorption, a potential desorption in the following 12 h. However, to confirm our hypothesis, more data are needed. Perrottet N. et al. [18] have confirmed the rapid efflux of ganciclovir from red blood cells into plasma, through permeation across the red blood cell membrane; this phenomenon could also play a role during blood filtration by CKRT and during hemoadsorption.

For amikacine, we observed low removal both at the Cmax and Cmin measurements, as already reported in vitro by Reiter [19]. We also observed a low impact of CS on ceftazidime removal. No previous data have been reported for this antibiotic but only for drugs belonging to the same class (third generation cephalosporine). Finally, among the ABs tested, levofloxacine resulted in the most removal by the CS, confirming that lipophilicity was the only pharmacokinetic factor moderately associated with CS clearance^25^ [17]. The AB kinetics during the study period were more stable with the hemofilter contribution in comparison to the CS cartridge, suggesting that a steady-state concentration during treatment was easily obtained with CKRT, whereas it may be more difficult to achieve with the CS column due to the cartridge exchange, the kinetics of the saturation in the first hours of treatment and the concentration-dependent effect of the removal of target molecules. Thus, in most of the categories of ABs studied in our population, CS’s clearance resulted in inferior results to that of the hemofilter. These data are very important, as higher doses of ABs have been suggested to guarantee PTA in patients undergoing CKRT [7], but clear indications for CS are not available. Indeed, this empirical approach may allow for adequate dosing even during hemoadsorption with the CS cartridge: in fact from our findings antimicrobials doses applied for CKRT have resulted adequate also with the simultaneous use of the hemoadsorption, without the need for further implementation.

We only found microbiological isolates in 5 out of 10 patients. In these patients, we evaluated the area under the curve (AUC) and PTA for different classes of antibiotics and found that PTA was achieved at each time point of the study period. The PK/PD targets adopted in this study were CSS = 4–6 X MIC for meropenem and ceftazidime, C*max* = 10 X MIC for levofloxacin and the ratio *Cmax*/MIC >8 for amikacin [15]. Conversely, the incidence of new-onset infections after 7 and 14 days during hemoadsorption showed a low incidence in our cohort (3 out of 10 after 7 days and 1 out of 10 after 14 days).

Our study presents some limitations. Firstly, we studied the pharmacokinetics of four antibiotics in a small cohort and no relevant statistical information could be inferred; therefore, our data need to be confirmed in larger populations. In addition, our pediatric population showed a mean age of 11.5 years, and our findings were very similar to what was already described in the adult population. Thus, from a future perspective, other categories of pediatric patients, such as infants and small children, should be investigated. Secondly, we monitored antibiotic levels irrespective of the CS changes; however, some authors [19] [20] reported a higher removal rate of drugs in the first hours of hemoadsorption treatment due to the saturation of the drug binding site on the absorber surface. Conversely, our study presents several strengths: we have assessed the role of a hemofilter and CS in extracorporeal clearance during routine clinical practice and not in a clinical trial. To achieve this aim, we collected blood samples at different points of the hemoadsorption circuits and analyzed the different contributions of the hemofilter and CS cartridge to antibiotic removal from the bloodstream. Moreover, for the first time, we have evaluated the impact of the CS cartridge on the capability of each administered antibiotic to reach the previously established PK/PD targets of efficacy.

## 4. Materials and Methods

### 4.1. Study Design

This prospective observational study was conducted between February 2021 and March 2023 in the PICU of Bambino Gesù Children’s Hospital, Rome, Italy. The study protocol was submitted to the local Ethics Committee and approved in January 2021 (protocol n° 144). Written informed consent was obtained from patients’ next of kin or guardian. Data presented were collected as part of a hospital audit on the safety of hemoadsorption with CS in critically ill children.

### 4.2. Data Collection and Characteristics of Treatment

Data were recorded using an electronic case report form (eCRF). Data collection at admission (enrolment) included demographics, comorbidities, etiologies of infection, site of infection, primary diagnoses, Pediatric Logistic Organ Dysfunction 2 score (PELOD-2) [21], vasoactive inotropic score (VIS) [22], Acute Kidney Injury (AKI) stage [23] and Acute Liver Failure [24,25]. Septic shock was defined according to the International Pediatric Consensus Conference and treated following the latest therapeutic guidelines [26]. AKI and its stage of severity was defined according to the KDIGO guidelines [23]. Acute Liver Failure (ALF) was defined as the abrupt onset of coagulopathy and biochemical evidence of hepatocellular injury, leading to rapid deterioration in liver cell function [25]. In order to evaluate the impact of CS on infectious status, we assessed any new onset of infections after 7 and 14 days from the start of hemoadsorption. Finally, mortality in PICU, mortality at 28 days, and the length of PICU stay were also recorded.

All critically ill children received ET based on a hemoadsorption treatment with CS in combination with CKRT. CKRT indications were AKI and/or fluid overload and/or electrolyte imbalance. CS was performed as a rescue therapy in children with proven or suspected diagnosis of septic shock in the context of inadequate response to standard therapy or refractory shock associated with multiple organ failure in the context of a cytokine storm syndrome.

### 4.3. CKRT and Hemoadsorption with CytoSorb

An 8 or 11.5 hemodialysis catheter was inserted into a central vein (internal jugular or femoral) as appropriate, according to the child’s size. CKRT was performed with a standard hemofilter (AN69ST) combined with CS continuous veno-venous hemodiafiltration (CVVHDF) or in continuous veno-venous hemofiltration (CVVH modality), using pre-filter reinfusion and an effluent dose of 2000 mL/h/1.73 m^2^. Blood flow was set based on the patient’s body weight (5–10 mL/kg/min for patients below 10 Kg, 5 mL/kg/min between 10 and 20 Kg and 100–150 mL/min for patients above 20 Kg). CS was inserted into the CKRT circuit in series with the hemofilter in a post-filter position; both the CKRT circuit and CS were flushed with a saline solution and primed with blood. Anticoagulation was managed with regional citrate anticoagulation with a starting citrate dose of 2.5 mmol/L and an aim of circuit calcium of 0.3–0.4 mmol/l and patient calcium of 1.1–1.25 mmol/L, or with unfractionated heparin with a continuous infusion of 10–20 UI/kg/h to achieve a post-filter activated clotting time (ACT) between 160 and 180 sec. CS therapy was continued on the basis of the clinical course as well as laboratory surrogates (lactate concentration and metabolic status including pH). CS was changed every 24 h, as recommended by the manufacturer.

### 4.4. Blood Samples Protocol

TDM of four ABs (meropenem, ceftazidime, amikacin and levofloxacin) was carried out throughout the whole study duration in each patient. Measurements for TDM have been performed from patient’s blood samples (venous central line or arterial line). In Table 3 the dosing regimens used for each AB are given.

**Figure 4:**
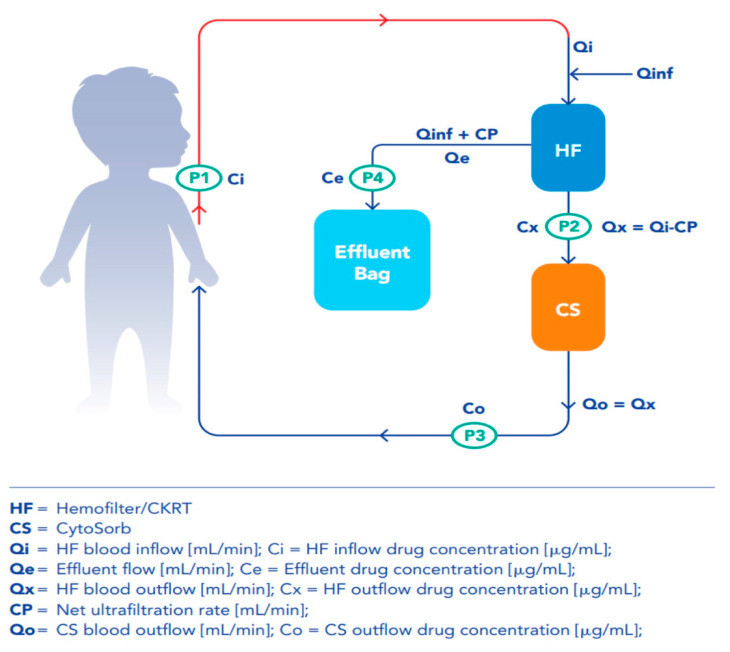
Graphical illustration of the extracorporeal circuit with blood sampling protocol. The 4 samplings in the patient and along the circuit were the patient’s blood sample (P1), hemofilter outflow = (CS cartridge inflow) (P2), CS cartridge outflow (P3) and effluent line (P4). In the lower section of the figure, the extracorporeal therapy flows are described: (1) hemofilter inlet = (Qin [mL/min]); (2) hemofilter outlet = (Qx [mL/min]) corresponding to CytoSorb inflow; (3) Net Ultrafiltration Rate = (CP [mL/min]); (4) effluent flow = (Qe [mL/min]); (5) CytoSorb outflow = (Qo [mL/min]).

In addition, in order to quantify the impact of the ET and the individual CKRT and CS contributions to AB removal, 3 sampling points for the determination of drug concentration were identified along the circuit: hemofilter outflow (CS cartridge inflow), CS cartridge outflow and effluent line (Figure 4). All samples for TDM were collected during a drug steady state. For time-dependent ABs, concentration trough (*Cmin*) was measured before the beginning of the next infusion in the patient’s blood sample (P1); drug concentration was also determined for each AB in blood samples simultaneously collected at each circuit point (P2, P3, P4). For concentration-dependent ABs, concentration max (C*max*) and C*min*, along with the concentration at each circuit point, were evaluated by collecting blood samples 1 h after the initiation of a bolus infusion and before the administration of the next dose, respectively. For time-dependent ABs, blood sampling was repeated for two to three consecutive days, whereas concentration-dependent AB samples were collected for two consecutive days. All blood samples were collected at the same time, irrespective of any change in the CS cartridge.

### 4.5. Clearance and Mass Removal Calculations

In order to analyze the effects of ET on the circulating drug levels, we considered the following parameters: ET Drug Clearance (CL [mL/min]), expressed as the total amount of blood purified from the administered drug *per* unit of time and ET Mass Removal (MR [µg/min]), calculated as the amount of drug removed over a unit of time. These parameters were used to calculate the total ET and the individual CKRT and CS contributions to the AB removal from bloodstream. CL and MR were calculated as follows in Table 4.

We have translated the mean clearance from milliliter per minutes (mL/min) to liter (L) per hours (h)( L/h) and the mean mass removal from microgram per minutes (µcg/min) to milligram (mg) per hour (h) (mg/h). The variables defining both parameters are reported in Figure 4. For each AB, CL and MR were calculated at C*min*. For the concentration-dependent ABs, both parameters were additionally calculated at C*min* and C*max*. For each AB, we calculated the Total Extracorporeal Clearance (CL_ET_) during the study period. CL_ET_ was assessed by considering the individual contributions of CKRT and CS. Residual renal clearance CL_ren_ was measured by urine collection during the monitoring period. For anuric patients CL_ren_ was assumed to be null. Data related to CS’s impact on overall clearance were also classified according available in vitro and in vivo data using similar cutoffs as low (<30%), moderate (30–60%) or high (>60%) removal potential [2].

### 4.6. Therapeutic Drug Monitoring

TDM of four ABs (meropenem, ceftazidime, levofloxacin and amikacin) was performed in the Laboratory of Metabolic Diseases and Drug Biology at Bambino Gesù Children’s Hospital in Rome.

For amikacin determination, liquid chromatography and mass spectrometry analysis were performed using an UHPLC Agilent 1290 Infinity II coupled to a 6470 Mass Spectrometry system (Agilent Technologies, Deutschland GmbH, Waldbronn, Germany), equipped with an ESI-JET-STREAM source operating in the positive ion (ESI+) mode. The software used for controlling this equipment and analyzing data was MassHunter Workstation (Agilent Technologies). The assay calibration curve was linear for amikacin and ranged from 1.28 to 58.8 µg/mL. Each batch of patients’ analyses included both low- and high-Quality Controls (QCs) at fixed concentrations of 3.70 and 39.4 µg/mL, respectively. Calibrators, QCs and patients’ samples were analyzed using a CE/IVD validated LC-MS/MS kit (FloMass Antibiotics) provided by B.S.N. S.r.l (Biological Sales Network, Castellone, Italy). Both the LC-MS/MS analytical kits included calibrators and QCs and were further validated according to European Medicines Agency (EMA) guidelines for bioanalytical method validation [27].

The UHPLC apparatus used for the determination of meropenem, ceftazidime and levofloxacin levels consisted of an Agilent 1290 Infinity II system equipped with a quaternary pump, a degassing line, a thermostated auto sampler, a column oven and a 10 μL cell DAD (Diode Array Detector) (Agilent Technologies). Specifically, the analyses of meropenem and ceftazidime levels were carried out by using a CE/IVD-validated HPLC kit (antibiotics in serum/plasma) provided by Chromsystems (Chromsystems Instruments & Chemicals GmbH, 82166 Grafelfing/Munich, Germany). QCs and patients’ samples were prepared according to the manufacturer’s instructions. Data were acquired and processed by using an OpenLAB Workstation (Agilent Technologies). Levofloxacin levels were determined by using a previously described method [28]. Briefly, 100 μL of plasma was spiked with 50 μL of Butylparaben (used as internal standard, IS) and vortexed for 5 s; thereafter, mixture was extracted with 250 μL of acetonitrile, mixed for 30 s and centrifuged at 13,000 rpm for 9 min. Supernatant was collected and evaporated under a liquid nitrogen flow. Reconstitution was achieved with 100 μL of a 2-(N-morpholino) ethanesulfonic acid (MES) buffer. The chromatographic run was realized on a Kinetex^®^ 2.6 μm EVO C18 100 × 2.1 mm column (Phenomenex, Torrance, CA, USA) thermostated at 50 °C with 0.5 mL/min flow. Analytes were discriminated by gradient elution. Mobile phase A consisted of Na_2_HPO_4_ × 2H_2_O 0.35% in H_2_O (adjusted to pH 7 with H_3_PO_4_), and mobile phase B was acetonitrile. Total running time was 8 min, and the injection volume was 5 μL. Levofloxacin concentrations were calculated from a linear calibration curve ranging from 1.0 to 25.0 µg/mL. For each analyzed compound, samples with drug concentrations above the higher calibration point were diluted and re-analyzed again.

### 4.7. Statistical Analysis

For descriptive data, continuous variables are expressed as mean ± standard deviation (SD) or median and interquartile range (IQR), according to their distribution; categorical variables are expressed as count (n) or percentage (%). All statistical analyses were performed using XLSTAT excel advanced statistical software (Version 2022.3, Addinsoft Inc, Paris, France) and NCSS 2021 Statistical Software (NCSS, LLC. Kaysville, UT, USA).

Comparisons between the different groups and over time were performed using non-parametric methods, such as the Mann–Whitney Test and Kruskal–Wallis Test for multiple group comparisons, as appropriate. Differences were considered statistically significant at *p* < 0.05.

## 5. Conclusions

To the best of our knowledge, this is the first study evaluating the clearance of meropenem, ceftazidime, levofloxacin and amikacin in critically ill children undergoing CKRT in combination with CS hemoadsorption. We found that the application of CS was associated with a lower clearance rate for meropenem, ceftazidime and amikacin but not for levofloxacin compared to the hemofilter. In the studied population, no significant clinical burden was observed, neither on ongoing infections nor on the occurrence of new infections, as a result of appropriate antibiotic coverage during treatments. However, more evidence is needed to confirm our data in a larger pediatric population.

## Figures and Tables

**Table 1:** Characteristics of the study population. Data are presented as medians and ranges or counts (n). HLH: Hemophagocytic Lymphohistiocytosis; AKI: Acute Kidney Injury. PELOD-2: Pediatric Logistic Organ Dysfunction-2. VIS: Vasoactive-Inotropic Score.

N = 10	
**Age**	11.5 (6.5–14.75)
**Male, n**	2 (10)
**Weight**	42 (26.25–71.25)
**Diagnosis on Admission**	
Severe sepsis, n	1 (10)
Septic shock, n	7 (10)
HLH, n	2 (10)
**Comorbidities**	
Liver disease, n	1 (10)
Pulmonary disease, n	1 (10)
Cardiovascular disease, n	1(10)
Hematologic disease, n	3 (10)
Neoplasia, n	2(10)
Other, n	4 (10)
**Etiologies of Infection**	
Gram positive, n	4 (10)
Gram negative, n	6 (10)
Virus, n	6 (10)
Fungus, n	1 (10)
Not detected, n	4 (10)
**Source of infection**	
Blood stream, n	2 (10)
Pulmonary, n	5 (10)
Abdominal, n	2 (10)
Other, n	2 (10)
**AKI stage**	
Stage III, n	10 (10)
**Acute Liver Failure**	
Yes, n	4(10)
Not, n	6 (10)
**PELOD-2**	9.5 (7.75–13.25)
**VIS**	75 (23.23–91.5)

**Table 2:** Average values and standard deviation (SD) of clearance and mass removal during the study period.

	Clearance Hemofilter (L/h) Qef × Ce/Ci × (Qi/ (Qi + Qe − CP)	Clearance CytoSorb (L/h): Qx × (Cx − Co)/Cx	Mass removal Hemofilter (mg/h) Qi × Ci – Qx × Cx	Mass Removal CytoSorb (mg/h) Qo × (Cx − Co)
**Meropenem**	1.1 (SD 0.3)	−1.5 (SD 1.5)	11.5 (SD 19)	−7.6 (SD 10)
**Ceftazidime**	2 (SD 1.3)	1.5 (SD 1.1)	61.8 (SD 52)	−4.1(SD 18.1)
**Amikacine** Cmax	1.5 (SD 0.9)	0.2 (SD 0.8)	26 (SD 43.7)	2.5 (SD 18.9)
**Amikacine** Cmin	1.5 (SD 0.3)	0.1 (SD 0.5)	1.7 (SD 1.4)	0.3 (SD 0.6)
**Levofloxacine** Cmax	1.8 (SD 0.8)	4.7 (SD 1.5)	22.5 (SD 3.1)	26 (SD 15.5)
**Levofloxacine** Cmin	1.7 (SD 1)	1.9 (SD 1.2)	0.6 (SD 5.9)	4.5 (SD 3.5)

**Table 3:** Reports on the number of patients per drug type and the dosing regimens adopted for the antibiotics tested in this study.

		n = 10
Number of Patients per Drug Type	Drug	Dosing
5	Meropenem	40 mg/kg q 8 h
3	Cefatazidime	50 mg/kg q 12 h
3	Levofloxacin	10 mg/kg q 12 h
2	Amikacin	15 mg/kg q 12 h

**Table 4:** Reports on extracorporeal therapies (ET), drug clearance (CL [mL/min]) and ET mass removal (MR [µg/min]) calculations. These parameters were used to calculate the total ET and the individual CKRT and CS contributions to the AB removal from bloodstream. HF = hemofilter; CS = CytoSorb; Qi = HF blood inflow (mL/min); Ci = HF inflow drug concentration (µg/mL); Qe = effluent flow (mL/min); Ce = effluent concentration (µg/mL); Qx = HF blood outflow (mL/min); Cx = HF outflow drug concentration (µg/mL); CP = Net Ultrafiltration Rate (mL/min); Qo = CS blood out flow (mL/min); Co = CS outflow drug concentration (µg/mL).

Parameter	ET CONTRIBUTIONS		
	Total ET	CKRT	CS
**CL**	Qef × Ce/Ci × (Qi/(Qi + Qe − CP) + Qx × (Cx − Co)/Cx	Qef × Ce/Ci × (Qi/(Qi + Qe − CP)	Qx × (Cx − Co)/Cx
**MR**	Qo × Co − Qi × Ci	Qx × Cx – Qi × Ci	Qo × (Co − Cx)

## Data Availability

All data analyzed and discussed in the framework of this study are included in this published article and its online supplementary information. The corresponding author may provide specified analyses or fully de-identified parts of the dataset upon reasonable request.

## Supplementary Materials

The following supporting information can be downloaded at https://www.mdpi.com/article/10.3390/antibiotics12091395/s1, Figure S1. Graphical representation of CytoSorb and hemofilter contributes to the Total Extracorporeal Clearance (CET). Table S1. Hypothetical pharmacodynamic target attainment (PTA) evaluated on the basis of clinical scenario and empirical antibiotic therapy.
