# Supplementary material for: Impact of Continuous Kidney Replacement Therapy and Hemoadsorption with CytoSorb on Antimicrobial Drug Removal in Critically Ill Children with Septic Shock: A Single-Center Prospective Study on a Pediatric Cohort
Source: Antibiotics (Basel). 2023 Aug 31;12(9):1395. doi: 10.3390/antibiotics12091395 (PMC10525765; doi:10.3390/antibiotics12091395)
Supplement: Supplementary file 1 [file antibiotics-12-01395-s001.zip › antibiotics-2554763-supplementary.pdf]

Supplementary Figure S1. Graphical representation of CytoSorb and hemofilter contributes to the Total Extracorporeal Clearance (CET). Light grey bars refer to CytoSorb's clearance contribution whereas dark grey bars refer to the hemofilter contribution. Data are expressed as percentage.

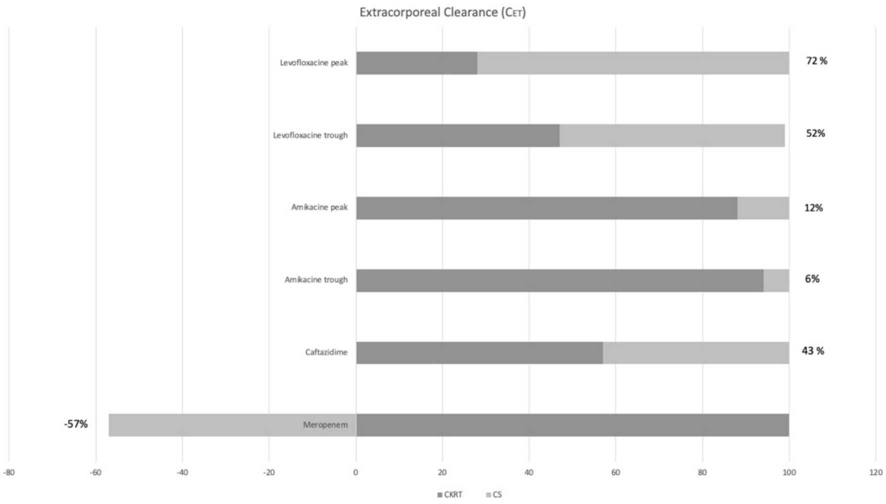

Supplementary Table S1. Hypothetical pharmacodynamic target attainment (PTA) evaluated on the basis of clinical scenario and empirical antibiotic therapy . MIC are referred to the EUCAST breakpoint tables.

CLINICAL SCENARIO	ANTIBIOTIC TREATMENT	**MIC breakpoints (mg/L)**	PTA	TDM RESULT
Septic Shock caused by Pseudomonas Aeruginosa	Meropenem	2	Css = 4–6 ×MIC	Meropenem Css 9-39 mcg/ml
Septic shock caused by Pseudomonas Aeruginosa	Ceftazidime	0.001	Css = 4–6 ×MIC	Ceftazidime . Css 29-37
Severe pneumonia caused by Staphylococcus aureus	Levofloxacin	0.001	Cmax 10 X MIC<br>C min < 3mg/L	Peak 6.2-4<br>Trough 2.7-2.2
Severe pneumonia caused by	Levofloxacin	0.001	Cmax 10 X MIC<br>C min < 3mg/L	Peak 17.7-4.5<br>Trough 3.8-0.9

Staphylococcus aureus				
Abdominal sepsis caused by Enterobacteriaceae	Meropenem	2	Css = 4–6 ×MIC	Meropenem Css 5.3-17 mcg/ml
Abdominal sepsis caused by Enterobacteriaceae	Amikacin	8	Cmax/MIC > 8–10 → Cmax 64-80 mg/dL	Peak 10.5-12.3 Trough 2.3-2.7
Abdominal sepsis caused by Enterobacteriaceae	Amikacin	8	Cmax/MIC > 8–10 → Cmax 64-80 mg/dL	Peak 18.8 29.7 Trough 1.9
